# Comparison of the effect of membrane sizes and fibre arrangements of two membrane oxygenators on the inflammatory response, oxygenation and decarboxylation in a rat model of extracorporeal membrane oxygenation

**DOI:** 10.1186/s12872-020-01581-3

**Published:** 2020-06-15

**Authors:** Fabian Edinger, Emmanuel Schneck, Charlotte Schulte, Johannes Gehron, Sabrina Mueller, Michael Sander, Christian Koch

**Affiliations:** 1 Department of Anaesthesiology, Operative Intensive Care Medicine and Pain Therapy, Justus Liebig University of Giessen, Rudolf-Buchheim-Strasse 7, 35392 Giessen, Germany; 2 Department of Adult and Pediatric Cardiovascular Surgery, Justus Liebig University of Giessen, Rudolf-Buchheim-Strasse 7, 35392 Giessen, Germany

**Keywords:** ECMO, Rat, Membrane oxygenator, Inflammation

## Abstract

**Background:**

Extracorporeal membrane oxygenation (ECMO) has gained widespread acceptance for the treatment of critically ill patients suffering from cardiac and/or respiratory failure. Various animal models have been developed to investigate the adverse effects induced by ECMO. Different membrane oxygenators have been used with varying priming volumes and membrane surfaces (Micro-1, small animal membrane oxygenator (SAMO)).

**Methods:**

Sixteen male Lewis rats (350–400 g) were randomly assigned to receive ECMO with Micro-1 or SAMO (*n* = 8, respectively). Venoarterial ECMO was established after cannulation of the femoral artery and the jugular vein. The cardiac output was measured using a left-ventricular conductance catheter. The oxygen fraction of the ECMO was set to 1.0, 0.75, 0.5 and 0.21 after a stabilisation period of 15 min. Further, arterial blood gas analyses were performed at baseline, and during the first hour every 15 min after commencing the ECMO, and subsequently every 30 min. Dilutional anaemia was calculated using haemoglobin concentration at baseline, and 15 min after the start of ECMO therapy. Moreover, inflammation was determined by measuring tumour necrosis factor alpha, interleukin-6 and -10 at baseline and every 30 min.

**Results:**

Animals of the Micro-1 group showed a significantly lower dilutional anaemia (ΔHaemoglobin t_0_ – t_0.25_: SAMO 6.3 [5.6–7.5] g/dl vs. Micro-1 5.6 [4.6–5.8] g/dl; *p* = 0.028). Further, significantly higher oxygen partial pressure was measured in the SAMO group, at an oxygen fraction of 0.75, 0.5 and 0.21 (380 [356–388] vs. 314 [263–352] mmHg, *p* = 0.002; 267 [249–273] mmHg vs. 197 [140–222] mmHg, *p* = 0.002; 87 [82–106] mmHg vs. 76 [60–79] mmHg, *p* = 0.021, respectively). However, no differences were found regarding the oxygen fraction of 1.0, in terms of carbon-dioxide partial pressure and cardiac output. Moreover, in the Micro-1 group tumour necrosis factor alpha was increased after 60 min and interleukin-6 after 120 min.

**Conclusion:**

While the dilutional anaemia was increased after commencing the ECMO, the oxygenation was augmented in the SAMO group. The inflammatory response was elevated in the Micro-1 group.

## Background

Extracorporeal membrane oxygenation (ECMO) was first performed by Gibbon et al. in 1954, during cardiac surgery [1]. The potential risks during cardiopulmonary bypass (CPB) with extracorporeal membrane oxygenation include cerebral stroke, inflammatory response and contact activation of the coagulation system [2, 3].Various CPB animal models have been described to investigate the adverse effects of CBP [4]. Large animal models of CPB – in lambs, pigs and dogs – have been established, but they are limited by personal requirements and high costs of operation and handling [4]. Thus, rodent models of CPB facilitate large sample sizes and can be conducted by a single experimenter. During CPB, cardiac arrest is often generated for cardiac surgery, and the lungs are not perfused. In contrast to CPB, the heart is still beating during ECMO, producing a continuous blood flow through the lungs, which prevents ischemia-reperfusion injuries.

The first description of a CPB model in rats was published by Popovic et al. in 1967 [5]. Subsequently, several modified models of CPB were established [6]. Most of them used oversized bubble or membrane oxygenators with large priming volumes [4]. Therefore, a priming of the extracorporeal circuit with the blood of donor rats was required. Further, investigations on the inflammatory response induced by extracorporeal oxygenation were compromised by the transfusion-donor blood [7]. In 2005 and 2006, two small rat membrane oxygenators with reduced priming volumes were developed. These oxygenators were used without the need for blood transfusion [8, 9]. The Micro-1 oxygenator (Micro-1, Kewei Rising Medical, Shenzhen, China) has a gas exchange area of 50 cm^2^ and a priming volume of 3.5 ml [8]. In contrast, the small animal membrane oxygenator (SAMO, M. Humbs, Valley, Germany) consists of a three-layered hollow-fibre membrane with a gas exchange area of 500 cm^2^ and a priming volume of 7 ml [9]. Both oxygenators have been used successfully in different models [8–15]. Models without a requirement for blood transfusion offer the opportunity to measure the extracorporeal oxygenation-induced inflammatory response. In addition to the priming volume, the oxygenation performance is an important parameter, which can be quantified by oxygen partial pressure (pO_2_).

The aim of our study was to examine the impact of different membrane sizes and fibre arrangements on inflammatory response, oxygenation and decarboxylation. Furthermore, we aimed to investigate the effect of the different priming volumes on the dilutional anaemia affecting the oxygen content (arterial saturation × haemoglobin × 1.34 + pO_2_ × 0.003) of the rat.

## Methods

### Animals

All procedures involving animals were conducted in compliance with the standards for animal care and were approved by the local committee for animal care (GI 20/26 G45/2018; Regierungspraesidium Giessen, Germany) [16].

Male Lewis rats (350–400 g), obtained from Janvier Labs (Le Genest St. Isle, France), were stored in conditions of 22 °C, 55% relative humidity and a day/night cycle of 14/10 h, with access to standard chow and water ad libitum. Rats were randomly divided into two groups to undergo ECMO with SAMO (*n* = 8) or Micro-1 (*n* = 8). Inclusion criteria contained body weight between 350 and 400 g. Unfortunately, two animals died during the surgical procedure and two animals had to be excluded due to severe blood loss during the surgical procedure. Therefore, four rats were replaced with additional animals.

### Anaesthesia and surgery

After inhalative induction of anaesthesia (5% isoflurane balanced with 100% oxygen), rats were intubated endotracheally (16 G cannula, B. Braun, Melsungen, Germany) and ventilated volume controlled (Harvard Inspira, Harvard Apparatus, Cambridge, UK) in a weight-adjusted manner. The animals were placed on an automated heating pad and a rectal temperature probe was inserted. Monitoring included end-tidal carbon-dioxide (CO_2_, MicroCapStar, CWE, Ardmore, Pennsylvania, USA), continuous electrocardiogram measurements, heart rate (HR), cardiac output (CO), stroke volume (SV), left-ventricular end diastolic volume (LVEDV), left-ventricular end diastolic pressure (LVEDP), left-ventricular ejection fraction (LVEF) and arterial blood pressure (systolic, diastolic and mean). Vascular accesses were placed surgically and consisted of the lateral tail vein for infusion and anaesthesia (24 G cannula, B. Braun, Melsungen, Germany), the tail artery for measurement of the arterial blood pressure and intermittent blood gas analysis (24 G B. Braun, Melsungen, Germany), the right femoral artery for ECMO inflow (22 G catheter, Terumo, Eschborn, Germany) and the right jugular vein for ECMO outflow (modified multi-orifice 17 G cannula, B. Braun, Melsungen, Germany). Further, a 2 F pressure–volume catheter (SPR-838, Millar, Houston, TX, USA) was inserted into the left ventricle through the right carotid artery for the continuous measurement of LVEDP and LVEDV.

### Extracorporeal membrane oxygenation

The ECMO circuit consisted of a venous reservoir (M. Humbs, Valley, Germany), a roller-pump (Verderflex Vantage 3000, Castleford, UK) and a membrane oxygenator. Both the SAMO and the Micro-1 oxygenator were used, depending upon the randomised groups. The oxygenators were primed with 250 IE Heparin (Ratiopharm, Ulm, Germany) and hydroxyethyl starch 6% (Voluven, Fresenius Kabi, Bad Homburg, Germany), according to the different priming volume of 7 ml (SAMO) and 3.5 ml (Micro-1).

The blood flow was initiated at a flow rate of 45 ml/kg/min, and continuously increased to 90 ml/kg/min. To measure the dilutional anaemia, arterial blood gas analyses – including haemoglobin concentration (Hb) – were performed just before and 15 min (min) after the start of ECMO therapy.

At first, ECMO commenced an oxygen fraction (FiO_2_) of 1.0. After a stabilization period of 15 min, the FiO_2_ was adjusted to 0.75, 0.5 and 0.21.

### Euthanasia at end of experiments

After the end of the experiments (t_2_) Isoflurane was adjusted at 5% and the blood flow rate on the ECMO was slowly decreased to 45 ml/kg/min and subsequently stopped. Accordingly, the rats were euthanised by exsanguination. Therefore, the whole blood was collected through the arterial and venous ECMO cannulas. Afterwards, the heart and lungs were resected.

### ELISA and blood gas analysis

Blood samples were collected at baseline (t_0_), every 15 min after starting the ECMO till t_1_ (60 min) and subsequently every 30 min till t_2_ (120 min). At each observation point, pO_2_, carbon-dioxide partial pressure (pCO_2_), Hb, pH, bicarbonate, base excess (BE), lactate, glucose, natrium, potassium, calcium and chloride were measured (ABL800, Radiometer, Copenhagen, Denmark). In addition, blood samples were centrifuged at 3000 g for 5 min and the plasma was stored at − 80 °C for further analysis at baseline (t_0_), and every consecutive 30 min.

To quantify the inflammatory response, the tumour necrosis factor alpha (TNF-α), interleukin (IL)-6 and IL-10 were measured by enzyme-linked immunosorbent assays (ELISA Kits R6000B, RTA00 and R1000 R&D System, Wiesbaden, Germany) according to manufacturer’s instructions. The probes were unfrozen only once.

### Statistics

The sample size calculation was performed using G*Power version 3.1.9.3 (Heinrich-Heine-Universität, Düsseldorf, Germany) and revealed a group size of nine animals with an alpha and beta error of 0.05. Further, the underlying effect size of 1.25 was calculated with Hb values from previous experiments. According to animal welfare regulations, intermittent statistical analyses were performed. Based on the regulations of the animal welfare board, the experiments were stopped due to statistically significant results after 16 animals. All data were expressed as the median, with an interquartile range (25th and 75th percentile). The Wilcoxon–Mann–Whitney test was used to compare the groups at the same time point. All statistical analyses were performed using SPSS Version 22 (IBM, Stuttgart, Germany). GraphPad Prism Version 7 was used to present the data (GraphPad Software, San Diego, CA, USA).

## Results

### Blood gas analyses

**Fig. 1:**
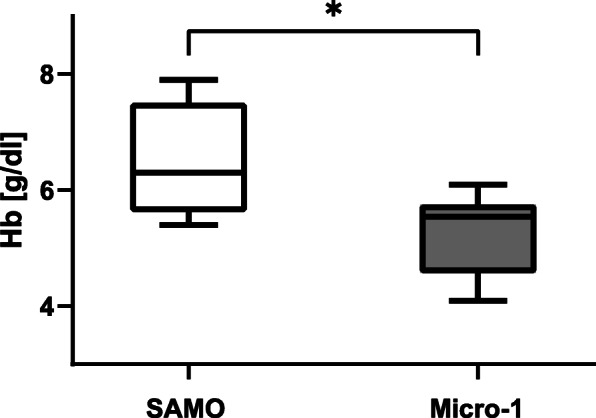
Dilutional anaemia. The dilutional anaemia was calculated using the Hb concentration at baseline (t_0_) and 15 min after commencing of the ECMO (t_0.25_). Values are expressed as medians with Min/Max values, * indicates that values are statistically significant different (*p* < 0.05), tested by using the Wilcoxon–Mann–Whitney test

The dilutional anaemia was calculated using the baseline Hb and the Hb value 15 min after commencing the ECMO. Animals in the Micro-1 group showed a significantly lower extent of dilutional anaemia (ΔHb t_0_ – t_0.25_: SAMO 6.3 [5.6–7.5] g/dl vs. Micro-1 5.6 [4.6–5.8] g/dl; *p* = 0.028; Fig. 1). Further measurements of the Hb revealed significant differences at t_0.25_ (Table 1).

**Table 1:** Blood gas analyses

Parameter	Group	Timepoints						
		t_0_	t_0.25_	t_0.5_	t_0.75_	t_1_	t_1.5_	t_2_
Hb	SAMO	13.6 [13.2–14.2]	6.8 [6.8–7.7] *	7.3 [6.9–7.5]	7.7 [7.2–7.7]	7.4 [7.2–7.7]	7.5 [7.3–7.6]	7.1 [6.7–7.5]
	Micro-1	13.4 [12.2–14.0]	7.9 [7.5–8.1]	7.7 [7.3–8.0]	7.5 [6.8–7.8]	7.4 [7.0–7.8]	7.2 [6.9–7.5]	6.9 [6.5–7.2]
pO_2_	SAMO	184 [115–196]	465 [418–498]	380 [356–388] **	267 [249–273] **	87 [82–106] *	247 [239–262] ***	241 [237–256] ***
	Micro-1	166 [128–197]	408 [394–475]	314 [263–352]	197 [140–222]	76 [60–79]	176 [122–194]	158 [104–189]
pCO_2_	SAMO	43 [39–48]	37 [36–38]	34 [31–35]	33 [31–35]	35 [33–36]	35 [32–36]	34 [32–37]
	Micro-1	40 [38–44]	36 [34–37]	34 [32–35]	33 [31–35]	33 [30–35]	33 [32–35]	35 [34–35]
pH	SAMO	7.40 [7.37–7.42]	7.44 [7.43–7.45]	7.49 [7.48–7.49] *	7.49 [7.47–7.51] *	7.48 [7.47–7.49] *	7.47 [7.46–7.49] **	7.46 [7.44–7.48] **
	Micro-1	7.40 [7.37–7.41]	7.45 [7.43–7.48]	7.47 [7.46–7.47]	7.45 [7.44–7.48]	7.47 [7.44–7.48]	7.43 [7.42–7.44]	7.41 [7.39–7.42]
Bicarbonate	SAMO	25.9 [24.0–26.7]	25.6 [24.2–26.4]	26.7 [24.2–26.4]	26.5 [24.7–27.9] *	26.5 [25.0–27.9] *	26.5 [24.6–27.5] *	24.8 [23.3–26.7] **
	Micro-1	24.8 [23.0–25.4]	25.1 [24.3–26.2]	25.2 [23.7–25.7]	24.3 [22.6–25.4]	24.0 [23.4–25.9]	23.0 [22–24.4]	22.1 [21.4–22.6]
BE	SAMO	1.7 [−0.6–2.6]	1.3 [− 0.4–2-2]	2.5 [−0.4–3.2]	2.3 [0.3–3.8] *	2.4 [0.6–3.8] *	2.4 [0.1–3.4] *	0.3 [− 1.3–2.6] **
	Micro-1	0.4 [−1.7–1.1]	0.7 [− 0.2–1.9]	0.8 [− 0.9–1.4]	−0.2 [− 2.1–1.1]	−0.5 [− 1.0–1.7]	−1.7 [− 2.9 – − 0.1]	−2.8 [− 3.6 – − 2.2]
Natrium	SAMO	141 [139–143]	141 [140–142]	141 [140–142]	141 [141–142]	142 [141–142]	142 [141–143]	141 [141–144]
	Micro-1	142 [141–144]	141 [140–141]	141 [140–142]	140 [139–142]	141 [140–142]	142 [141–142]	142 [140–143]
Potassium	SAMO	3.8 [3.6–4.0]	3.7 [3.6–3.9]	3.9 [3.8–4.2]	4.0 [3.8–4.2]	3.9 [3.8–4.2]	3.9 [3.7–4.1]	4.0 [3.9–4.2]
	Micro-1	3.9 [3.8–4.0]	3.7 [3.6–3.8]	4.1 [3.9–4.3]	4.1 [4.0–4.2]	4.0 [3.9–4.1]	4.1 [3.8–4.3]	4.2 [4.1–4.3]
Calcium	SAMO	1.41 [1.37–1.44]	1.47 [1.44–1.48]	1.44 [1.42–1.46]	1.44 [1.43–1.48]	1.45 [1.44–1.48]	1.45 [1.43–1.48]	1.46 [1.44–1.50]
	Micro-1	1.43 [1.41–1.46]	1.45 [1.44–1.47]	1.47 [1.46–1.49]	1.46 [1.42–1.49]	1.47 [1.46–1.48]	1.47 [1.45–1.48]	1.48 [1.47–1.52]
Chloride	SAMO	106 [103–107]	108 [108–109]	108 [107–111]	108 [106–108]	108 [107–109]	108 [107–109]	109 [108–109]
	Micro-1	107 [105–108]	108 [107–109]	109 [108–110]	108 [108–110]	109 [107–111]	109 [108–110]	109 [108–110]
Glucose	SAMO	165 [154–172]	153 [143–163]	128 [118–138]	123 [116–141]	129 [121–138]	127 [118–135]	118 [111–125]
	Micro-1	176 [154–181]	163 [161–169]	133 [118–135]	122 [112–149]	122 [120–139]	121 [113–125]	112 [111–119]
Lactate	SAMO	2.1 [1.6–2.2]	1.8 [1.5–2.3]	1.8 [1.6–2.1]	1.5 [1.3–1.7]	1.4 [1.0–1.5]	1.6 [1.4–1.8]	1.6 [1.4–1.8]
	Micro-1	1.9 [1.5–2.0]	1.8 [1.5–2.1]	1.6 [1.4–1.7]	1.2 [1.1–1.5]	1.2 [1.0–1.5]	1.6 [1.4–1.9]	1.5 [1.4–1.9]

Results of the blood gas analyses are presented as the median with an interquartile range (25th and 75th percentile)

*Abbreviations*: *SAMO* Small animal membrane oxygenator, *Micro-1* Micro-1 rat oxygenator, *Hb* Haemoglobin, *pO*_*2*_ Oxygen partial pressure, *pCO*_*2*_ Carbon-dioxide partial pressure, *BE* Base excess

Asterisks display the degree of statistical significance: *: *p* < 0.05; **: *p* < 0.01; ***: *p* < 0.001

**Fig. 2:**
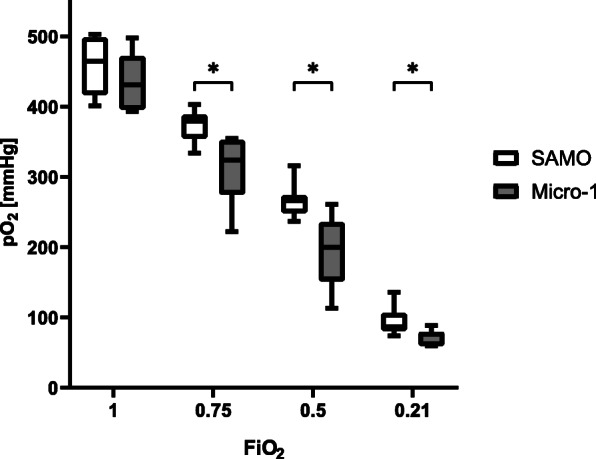
Oxygen partial pressure. ECMO began with FiO_2_ at 1.0, and the pO_2_ was measured after 15 min – the FiO_2_ was adjusted to 0.75, 0.5 and 0.21 respectively. Values are expressed as medians with Min/Max values, * indicates that values are statistically significant different (*p* < 0.05), tested by using the Wilcoxon–Mann–Whitney test

Moreover, SAMO reached significantly higher pO_2_ values at different FiO_2_ (Fig. 2). After t_1_ the FiO_2_ was adjusted to 0.5 and significantly higher pO_2_ were measured in the SAMO group at t_1.5_ and t_2_ (Table 1).

In addition, no significant differences between both groups were measured regarding the pCO_2_. The pH-value was significantly higher in the SAMO group between t_0.5_ and t_2_ (Table 1). Accordingly, SAMO resulted in increased bicarbonate levels and BE at t_0.75_, t_1_, t_1.5_ and t_2_ (Table 1).

Measurements of natrium, potassium, calcium, chloride, glucose and lactate revealed no differences between the two groups (Table 1).

### Measurement of inflammatory parameters

**Fig. 3:**
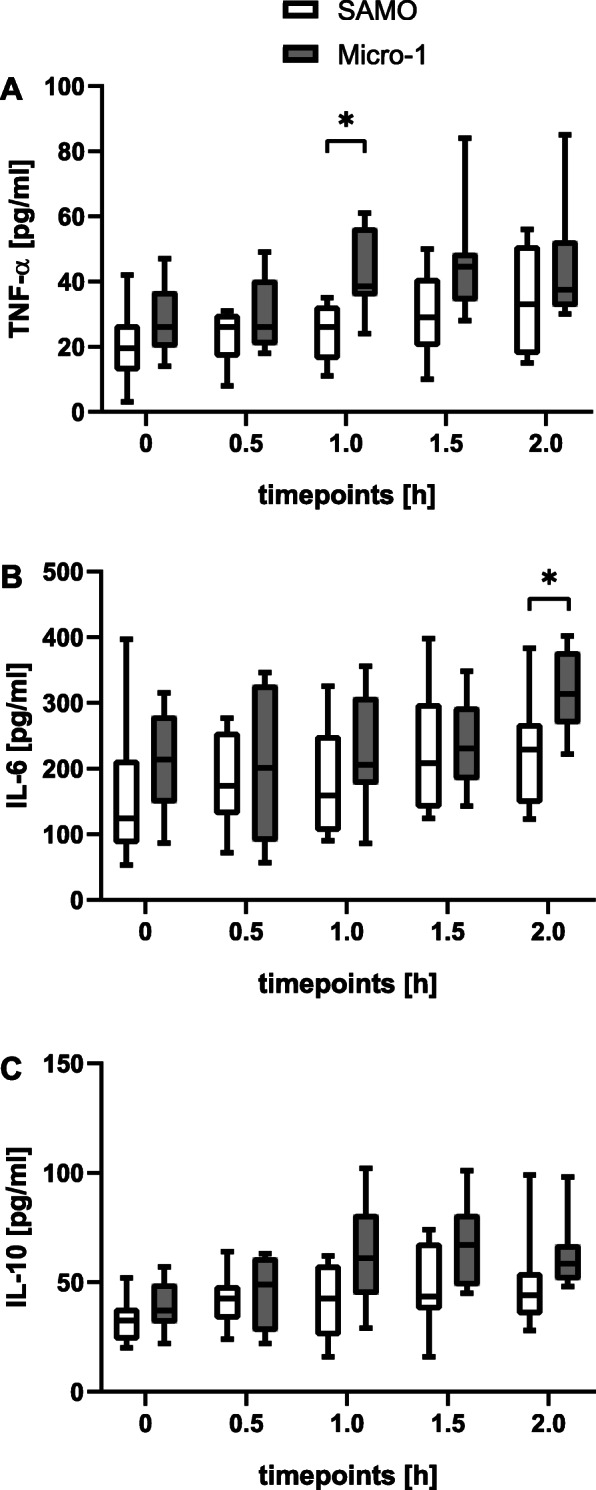
ECMO-induced inflammation. **a** TNF-α, **b** IL-6 and **c** IL-10 were measured at baseline (t_0_) and every 30 min after commencing the ECMO (t_0.5_, t_1_, t_1.5_ and t_2_). While TNF-α and IL-6 reflect the proinflammatory response, IL-10 indicates the anti-inflammatory reaction. Values are expressed as medians with Min/Max values, * indicates that values are statistically significant different (*p* < 0.05), tested by using the Wilcoxon–Mann–Whitney test

The TNF-α levels were significantly higher in the Micro-1 group at t_1_ (SAMO 26 [16–32] pg/ml vs. Micro-1 39 [36–56] pg/ml, *p* = 0.005; Fig. 3). Moreover, the serum values of IL-6 were significantly increased in the Micro-1 group at t_2_ (SAMO 229 [147–269] pg/ml vs. Micro-1 314 [268–378] pg/ml, *p* = 0.028; Fig. 3). Further analysis, at other times, for TNF-α and IL-6, as well as the measurements of IL-10 did not reveal significant differences between the two groups.

### Haemodynamic measurements

**Fig. 4:**
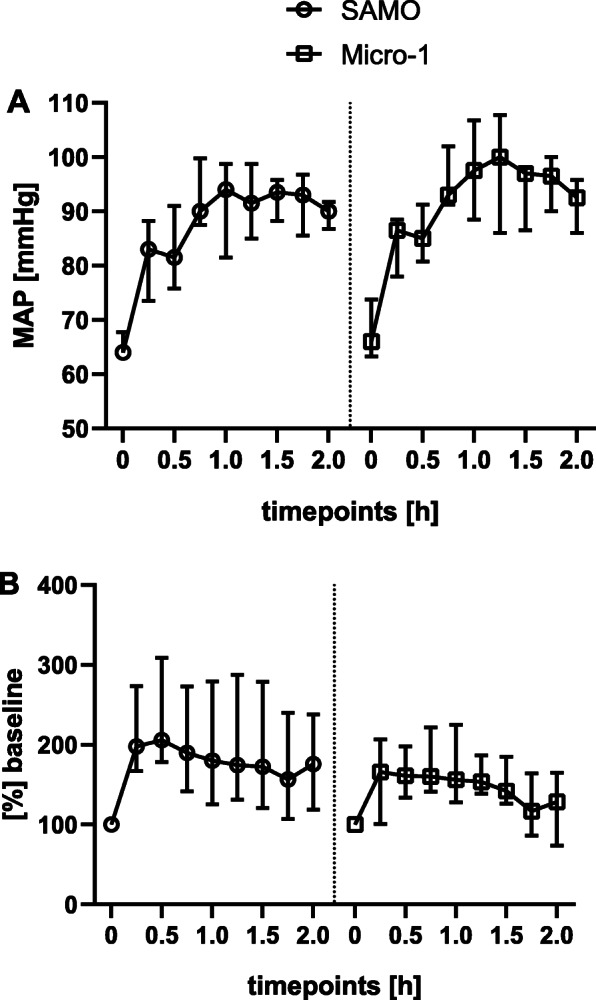
Hemodynamic monitoring. **a** MAP and **b** CO were continuously measured and analysed at baseline (t_0_), and every 15 min after start of the ECMO. Values are expressed as medians with an interquartile range, * indicates that values are statistically significant different (*p* < 0.05), tested by using the Wilcoxon–Mann–Whitney test

Haemodynamic values were analysed every 15 min after commencing the ECMO during a two-hour observation period. No significant differences in HR, diastolic and mean arterial blood pressure were found between the two groups (Fig. 4). The systolic blood pressure was increased in the Micro-1 group at t_1.75_ and t_2_ (t_1.75_ SAMO 122 [118–132] mmHg vs. Micro-1132 [129–141] mmHg, *p* = 0.038; t_2_ SAMO 122 [120–125] mmHg vs. Micro-1126 [123–130] mmHg, *p* = 0.038).

Furthermore, measurements of CO, SV, LVEDV, LVEDP and LVEF did not reveal significant differences between the SAMO and Micro-1 group (Fig. 4).

## Discussion

This study compared two different membrane oxygenators (SAMO and Micro-1) in in-vivo rat models of ECMO. Hemodynamic measurements, oxygenation, haemodilution and ECMO-induced inflammation were investigated.

In the past, both oxygenators have been used in different rat models, including models of CPB and deep hypothermic cardiac arrest (DHCA) [8, 9, 12–15, 17]. To the best of our knowledge, they have not been compared regarding their oxygenation, decarboxylation, dilutional anaemia and inflammation response to date.

The haemoglobin concentration just after commencing the ECMO was significantly lower in the SAMO group, reflecting the different priming volumes of both oxygenators (SAMO 11 ml vs. Micro-1 9 ml). Moreover, the pO_2_ was significantly increased in the SAMO group, which could be caused by the larger membrane surface (SAMO 500 cm^2^ vs. Micro-1 50 cm^2^). After two-hours of ECMO therapy, the pO_2_ was significantly reduced in the Micro-1 group, suggesting the SAMO is more long-term stable. However, there was no difference in decarboxylation between both groups. Magnet et al. used the SAMO in a rat model of cardiac arrest. After 15 min of ECMO with a blood flow of 100 ml/kg/min and a FiO_2_ of 1.0, they measured a pO_2_ of 443 mmHg, which is similar to that in our findings [13]. The Micro-1 oxygenator was validated in a rat model of CPB by Dong et al. [8]. A pO_2_ of 286 ± 21 mmHg was measured with a blood flow of 100–150 ml/kg/min and a FiO_2_ of 1.0, which was lower than that found in our measurements (Micro-1 pO_2_ 408 [394–475] mmHg). While the ventilation was continued during ECMO, Dong et al. stopped the mechanical ventilation of the lungs during CPB, which might be the cause of this difference.

Further, the pCO_2_ was adjusted by the sweep gas flow over the membrane, which explained that the pCO_2_ was reported between 35 and 45 mmHg in previous studies [8, 13, 17].

The extracorporeal circuit, which consists of a venous reservoir, a roller pump, a membrane oxygenator and several tubes, must be primed. Further, the priming volume varies between 8 and 15 ml reflecting the different oxygenators, reservoirs and tubes [10, 11, 15]. For this reason, dilutional anaemia occurs after commencing the extracorporeal circulation. The reported haematocrit values during extracorporeal circulation vary between 22 and 32% [12, 13, 17]. In our model, haematocrits of 21% (SAMO) and 24% (Micro-1) were measured. Moreover, the blood volume of the rat also affects the dilutional anaemia and can be calculated by the following formula: blood volume (ml) = bodyweight (g) × 0.06 + 0,77 [18]. The body weight of the rats used in literature on the topic vary between 300 g and 500 g, resulting in blood volumes between 19 ml and 31 ml [13, 15]. We used rats weighing 350–400 g to prevent heart failure, a phenomenon that also occurs more frequently in old animals.

Extracorporeal circulation causes systemic inflammation. Mechanical cell damage and contact with the extracorporeal circuit, especially with the membrane of the oxygenator, are assumed to be the cause of this [2]. The systemic inflammatory response syndrome like cascade includes the intrinsic and extrinsic coagulation pathway, complement system and endothelia cells, leukocytes, platelets and cytokines [2]. Other factors influencing the inflammatory response include the health of the animals and the surgical trauma induced by vascular access. Cannulation with an open chest procedure leads to an aggravation of the inflammatory response. Therefore, we chose a vascular access procedure without sternotomy.

Interestingly, we observed higher levels of the proinflammatory cytokines TNF-α and IL-6 in the Micro-1 group, although the membrane surface is smaller than that of the SAMO group (SAMO 500 cm^2^ vs. Micro-1 50 cm^2^). The Micro-1 oxygenator was gas sterilized prior to use and applied only once. In contrast, the SAMO consists of a plexiglass chassis, which was reused and cleaned with an enzyme cleaner (Helizyme, B. Braun, Melsungen, Germany), and a three-layer hollow-fibre membrane – applied only once – was also gas sterilized prior to use.

During CPB, the heart and the lungs are not regularly perfused, resulting in an ischemia-reperfusion injury [19]. Therefore, our approach was to compare the SAMO and Micro-1 groups during ECMO with continuous ventilation and blood flow through heart and lungs to avoid ischemia and reperfusion. Hence, our results cannot be applied to other rat models using the SAMO or Micro-1 oxygenator during CPB or DHCA.

Furthermore, it is important to note the limitations of our work: first, the surgical vascular access results in a distal ischemia caused by vascular ligation. Furthermore, the commencement of the ECMO caused a dilutional anaemia with low haemoglobin concentrations. No elevation in lactate was measured, suggesting that the oxygen delivery was greater than the oxygen consumption. Second, the observation period during ECMO was only 2 h. Last, despite the sample size calculation, the study cohort of eight animals per group remains small.

Besides, shorter tubes could have led to reduced priming volumes with less dilutional anaemia. Further experiments should use SAMO or Micro-1 oxygenators regarding the underlying scientific question.

## Conclusion

Herein, we compared the SAMO and Micro-1 oxygenators in terms of oxygenation, decarboxylation, dilutional anaemia and inflammation response. While the Hb concentration was impaired after commencing the ECMO, the oxygenation was increased in the SAMO group. Further, the proinflammatory cytokines – TNF-α and IL-6 – were elevated in the Micro-1 group.

In summary, the Micro-1 oxygenator induced a higher extent of inflammatory response, had a lower oxygenation capacity and showed less dilutional anaemia. Contrarily, the use of SAMO resulted in a weaker inflammatory reaction, higher oxygenation capacity and increased dilutional anaemia. According to our findings, further studies on ECMO-induced inflammation should be performed using the Micro-1 oxygenator, while oxygenation studies should be investigated with the SAMO.

## Data Availability

The datasets used and analysed during the current study are available from the corresponding author upon reasonable request.

## Abbreviations

BE: Base excess
CO: Cardiac output
CPB: Cardiopulmonary bypass
DHCA: Deep hypothermic cardiac arrest
ECMO: Extracorporeal membrane oxygenation
FiO_2_: Oxygen fraction
Hb: Haemoglobin
HR: Heartrate
IL-10: Interleukin-10
IL-6: Interleukin-6
LVEDP: Left ventricular end diastolic pressure
LVEDV: Left ventricular end diastolic volume
LVEF: Left ventricular ejection fraction
MAP: Mean arterial pressure
min: Minutes
pCO_2_: Carbon-dioxide partial pressure
pO_2_: Oxygen partial pressure
SAMO: Small animal membrane oxygenator
SV: Stroke volume
TNF-α: Tumor necrosis factor alpha
